# Assessment of Seroprevalence and Associated Risk Factors for Anaplasmosis in *Camelus dromedarius*

**DOI:** 10.3390/vetsci9020057

**Published:** 2022-01-30

**Authors:** Roua A. Alsubki, Fatima M. Albohairy, Kotb A. Attia, Itoh Kimiko, Abdelfattah Selim, Mohamed Z. Sayed-Ahmed

**Affiliations:** 1 Department of Clinical Laboratory Science, College of Applied Medical Sciences, King Saud University, P.O. Box 2455, Riyadh 11451, Saudi Arabia; ralsubki@ksu.edu.sa (R.A.A.); fmalbohairy@pnu.edu.sa (F.M.A.); 2 Department of Biochemistry, College of Science, King Saud University, P.O. Box 2455, Riyadh 11451, Saudi Arabia; 3 Institute of Science and Technology, Niigata University, Niigata 950-2181, Japan; kimiko.itoh@gmail.com; 4 Department of Animal Medicine (Infectious Diseases), Faculty of Veterinary Medicine, Benha University, Toukh 13736, Egypt; 5 Department of Internal Medicine and Infectious Diseases, Faculty of Veterinary Medicine, Mansoura University, Mansoura 35516, Egypt; drzakaria-infect@hotmail.com

**Keywords:** anaplasmosis, cELISA, risk factors, camels, Egypt

## Abstract

(1) Background: Anaplasmosis is an infectious disease in camels caused by an obligate intracellular bacterium that is transmitted by ticks. (2) Methods: A cross-sectional study was conducted during 2020 to study the seroprevalence of *Anaplasma* spp. among *Camelus dromedarius* in three governorates in Egypt and assess the associated risk factors. Serum samples from 365 camels were examined by a competitive enzyme-linked immunosorbent assay (cELISA) test. (3) Results: Overall, the seroprevalence of anaplasmosis among camels was 18.6%. Multivariable logistic regression was performed, and it was discovered that tick infestation, application of acaricides, grooming practice and body condition were potential risk factors for *Anaplasma* spp. infection (odds ratio > 1) in dromedary camels. In contrast, the locality in which the camels lived and their age were not significant effects with regard to the occurrence of anaplasmosis. (4) Conclusions: The current findings suggest that improvement of protective measures to limit the effects of the identified risk factors can help to reduce the spread of anaplasmosis among camels in Egypt.

## 1. Introduction

The camel is a multipurpose animal that lives in arid and semi-arid areas. In many countries, including Egypt, the dromedary (*Camelus dromedarius*), often known as the one-humped camel or Arabian camel, is a very valuable species. Camels are of socioeconomic importance in Egypt since they can be utilized as sources of meat and milk and as a mode of transportation and tourist rides. Moreover, Camel milk is a healthy food for people since it contains more vitamin C and has less cholesterol [1]. Camels outperform farmed cattle due to their unique physiological characteristics that allow them to survive lengthy periods of time without access to water [2]. Despite their vast resources, camels can get various infectious diseases that affect their health and productivity [3,4,5,6,7].

Many animals, including camels, are affected by haemoparasites. The primary vectors for the transmission of these pathogens are ticks. Many studies have been conducted on tick-borne pathogens in camels, including trypanosomiasis, theileriosis, babesiosis and anaplasmosis [8,9,10].

Anaplasmosis is a vector-borne disease of ruminants [11,12,13]. Several varieties of *Anaplasma* species can infect camels, such as *Anaplasma centrale* (*A. centrale*), *Anaplasma marginale* (*A. marginale*), *Anaplasma phagocytophilum* (*A. phagocytophilum*) and *Anaplasma platys* (*A. platys*) [14]. *Anaplasma* is an obligate intracellular bacterium that belongs to the order *Rickettsiales, Anaplasmataceae* family, and is transmitted biologically and mechanically by hard ticks such as *Ixodes ricinus*, *Dermacentor* spp., *Rhipicephalus* spp. and *Boophilus* spp. [15,16]. *Anaplasma* spp. are transmitted both biologically and mechanically [16]. In Egypt, most of the hard tick species infesting camels belong to *Hyalomma*, *Haemaphysalis*, *Amblyomma* and *Rhipicephalus* [17]. At the same time, *Hyalomma anatolicum excavatum* and *Boophilus annulatus* were found on cows [18].

In camels, anaplasmosis usually appears as a subclinical infection or as a co-infection. However, it can manifest clinically as fever, anaemia, emaciation, slight ataxia, anorexia, jaundice or enlargement of the lymph nodes [3,19].

Clinical diagnosis of these organisms is difficult due to the nonspecific clinical indications [20]. As a result, in disease-endemic areas, care is important, as well as appropriate diagnostic tests to aid in infection confirmation.

Routine diagnosis in the laboratory for direct detection of anaplasmosis in camels depends mainly on microscopic examination. Light microscopy is the cheapest and fastest laboratory test, but it is a low sensitive technique, and it is heavily reliant on examiner skill [21]. Moreover, the efficacy of this method is affected by the time interval that passes between the onset of the clinical signs of disease and the collection of the sample. This delay leads to the unreliability of this method in carrier animals [3].

In both laboratory and field research, nucleic acid-based techniques such as loop-mediated isothermal amplification (LAMP), polymerase chain reaction (PCR) and quantitative real-time PCR (qPCR) have been used to detect *Anaplasma* infection [22]. However, the sensitivity of these techniques is limited, especially in persistently infected animals characterized by low-level bacteremia [23].

On the other hand, serological assays have advantages for the investigation of antibodies in infected animals at all stages of the *Anaplasma* infection [24]. In addition, serological tests are preferred to identify previous exposure to the pathogens as well as carrier animals. The most common of these tests is the indirect fluorescent antibody technique (IFAT); however, the enzyme-linked immunosorbent assay (ELISA) is reliable and convenient and offers more advantages than IFAT [25,26,27].

Anaplasmosis has been reported in dogs, cattle, water buffalo, camels and humans in several localities of Egypt [28,29,30,31,32,33]. Nonetheless, there is a lack of frequent monitoring and control procedures in the field. Moreover, *A. marginale* is most commonly seen in cattle, camels, and arthropods that live on a variety of host animals [34]. In camels, anaplasmosis has been reported in several parts of Egypt, such as Assuit, South Sinai, Matrouh and Luxor. These findings were based on microscopic examination and use of cELISA, IFAT and polymerase chain reaction (PCR) tests [25,35]. Recently, a study detected antibodies against *Anaplasma* spp. in camels from Egypt based on commercial cELISA kits that showed 100% sensitivity and specificity [4]. However, few studies have focused on the risk factors that are associated with *Anaplasma* spp. infection in camels.

Therefore, the present study aimed to determine the seroprevalence of *Anaplasma* spp. in camels and evaluate the associated risk factors for *Anaplasma* spp. infection.

## 2. Materials and Methods

### 2.1. Ethics Statement

The ethical committee for Animal Experiment of the faculty of veterinary medicine, Benha University, approved all procedures involving the handling and collection of samples from camels used in this study. The camel’s owners gave their verbal approval for the samples to be collected.

### 2.2. Study Area

**Figure 1:**
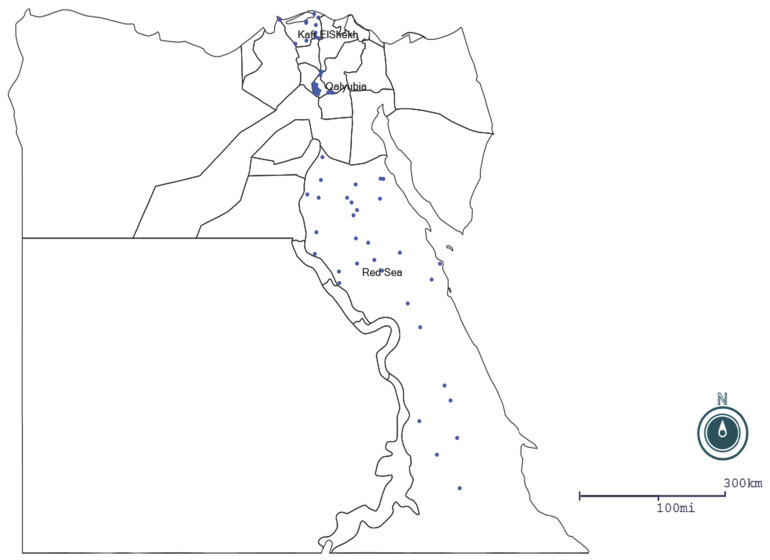
Geographical areas that were visited in the study and number of positive samples represented by blue dots. MAP generated by EPI MAP (CDC).

A cross-sectional study was conducted during 2020 in three governorates of Egypt that had high camel populations. The study areas were the governorates of: Qalyubia (30°25 N to 31°13 E), Kafr ElSheikh (31.1107° N, 30.9388° E) and the Red Sea (25°32′1″ N 33°26′18″ E) (Figure 1). The climatic conditions of Qalyubia and Kafr ElSheikh governorates are wet winters with moderate rainfall and dry summers, while the Red Sea area has a desert climate during the whole year with virtually no rainfall. These warm climatic conditions are suitable for tick propagation. Ticks are the principal vector for transmission of *Anaplasma* spp. Moreover, the types of observed ticks in examined camels in the study areas were mainly *Rhipicephalus annulatus, Hyalomma dromedarii* and *Rhipicephalus turanicus* [17].

### 2.3. Sample Collection and Preparation

The required sample size of the study was calculated according to a formula devised by Thrusfield [36] as follows:

$$n=\frac{{\left(1.96\right)}^{2\;}Pexp\;\left(1-Pexp\right)}{d^{2}}$$

In which *n* is the sample size, *Pexp* is the expected prevalence rate and *d* is precision. The expected prevalence rate that was used in this study was 34.1%, as previously reported by Parvizi et al. [4], with a 95% confidence interval and 5% precision. The majority of the study animals were chosen at random from small-scale farmers that keep camels as working animals. A total of 365 blood samples were collected from the jugular vein of camels using a vacuum tube without EDTA. The collected blood samples were transferred in iceboxes to the Veterinary Diagnostic Laboratory, Faculty of Veterinary Medicine, Benha University. The sera were collected using clean, sterile vacuum tubes and were separated by centrifugation at 3500× *g* for 10 min. The examined camels were categorized according to their locality (Qalyubia, Kafr Elsheikh or the Red Sea), sex (male or female) and age (≤2, >2–5 and >5 years old). Moreover, information regarding tick infestation, whether or not acaricides had been applied (trimonthly application), grooming practice (removing thick hair that has accumulated grain, grime, and mats on a regular basis) and body condition (emaciated, decrease the bodyweight than normal or healthy) was collected to investigate their association to infection.

### 2.4. Serological Analysis

The specific antibodies against *Anaplasma* spp. were investigated in the collected sera through the use of a commercial competitive ELISA (cELISA) v2 (VMRD Inc, Pullman, WA, USA), which is able to detect antibodies against the major surface protein 5 (MSP5) of *A. marginale*, *A. centrale* and *A. ovis* [37]. The process of the test was performed according to the guidelines of the manufacturer. This kit had previously been validated to show 100% sensitivity and specificity in the detection of *Anaplasma* spp. antibodies in camels [4]. The sample was considered positive if the cut-off value (Ct) was equal to 0.42.

### 2.5. Statistical Analysis

Data regarding the anaplasmosis surveillance were analyzed by use of the statistical program for the social sciences (SPSS) software v24 (IBM SPSS Inc., Chicago, IL, USA). The data were verified through the use of the chi-square test, and the results were considered significant if *p* < 0.05. The results were analyzed through the use of univariable logistic regression to evaluate the association between each variable and prevalence of *Anaplasma* spp. The Hosmer–Lemeshow goodness-of-fit test was applied to evaluate the fit of the multivariable logistic regression model. The variables with *p* ≤ 0.2 were included in the multivariable regression model, which was used to determine the risk factors, odds ratios (ORs) and confidence intervals (CIs) of each significant variable in the univariable analyses. Odds ratios of >1 suggested an increased risk of seroprevalence of anaplasma infection, whereas odds ratios of <1 suggested a decreased risk of seroprevalence of anaplasma infection.

## 3. Results

The present study demonstrated an overall 18.6% (68/365) seroprevalence of anaplasmosis among camels that lived in the three investigated areas. The highest seroprevalence for *Anaplasma* spp. in camels was estimated to be in the Red Sea governorate (21.3%, *n* = 32) (Table 1). In order of increasing magnitude, the seroprevalence was 13% (*n* = 13) and 20% (*n* = 23) in Kafr ElSheikh and Qalyubia governorates, respectively (Table 1).

According to the univariable analysis, seropositivity to *Anaplasma* spp. in camels was associated significantly with female sex, tick infestation, non-application of acaricides, poor grooming practice and poor body condition (*p* < 0.005). The highest seroprevalence rates were observed in females (21.5%), infested camels with ticks (33%) and in cases of the absence of acaricides application (23.5.%) and grooming application (25.9%), Table 1.

In addition, a strong association was found between animals in an emaciated condition and *Anaplasma* spp. infection. On the other hand, there was no significant interaction between age and *Anaplasma* spp. infection (Table 1).

Significant variables that were obtained through univariable studies were then analyzed multivariably. The animal’s age and locality were removed as factors. In this study, it was found that females were two times more likely to be infected than males (95% CI: 0.91–4.35). Furthermore, tick infestation of camels (OR = 1.12, 95% CI: 0.54–2.32), lack of acaricide application (OR = 1.02, 95% CI: 0.39–2.68), absence of grooming (OR = 1.3, 95% CI: 0.53–3.18) and an emaciated condition of the examined camels (OR = 9.36, 95% CI: 4.36–20.10) were found to be potential risk factors for *Anaplasma* spp. infection in camels (Table 2).

## 4. Discussion

Dromedary camels can harbor a variety of pathogens, including *Anaplasma*. This genus has been reported in the last few years in some studies, but the epidemiological data remains limited.

A description of the epidemiological status of anaplasmosis and evaluation of the risk factors that are potentially related to disease in camels helps to improve the understanding of the dynamics of and potential control methods for the disease [38].

In this study, the antibodies against *Anaplasma* spp. in camels were detected in 68 of 365 animals, and the seroprevalence rate was recorded as 18.6%. Despite the large differences in bioclimatic features between the three sites studied, the prevalence rates do not differ significantly (*p* > 0.05). The highest rate, 21.3%, was observed in the Red Sea, while the lowest rate (13%) was found in the Kafr ElSheikh governorate. This is likely due to the frequent movement of camels between these areas and the similarities of tick populations infesting camels in the sampling locations [39]. The high rate observed in the Red Sea governorate may be due to the nature of this area, which is a border governorate that receives camels from neighbouring countries. These camels may be carriers of haemoparasites. In addition, different humidity levels that enable the proliferation of vectors and transmission of *Anaplasma* can affect the prevalence rate [40].

In Egypt, recent studies revealed 47.4%, 47.4% and 67.37% prevalence rates of anaplasmosis in camels. These findings were based on tests that employed cELISA, microscopic examination and PCR techniques [3,4]. Other studies conducted in various countries have reported high prevalence rates of 26–95.5%, 34.2%, 39.6% and 61.11% in Saudi Arabia, Iran, Morocco and Niger, respectively [14,41,42,43]. However, other studies have reported low prevalence rates of anaplasmosis that ranged from 6% to 13.33% [44,45,46,47].

The differences in prevalence rates may be attributable to sample numbers, the diagnostic techniques were used, demographics of the research locations and disease endemicity in each study region [46]. Furthermore, tick control programmes, farm management, husbandry practises, wildlife reservoir hosts and/or abiotic variables may all play a role in the large disparity in prevalence rates. Several studies have found that the incidence of *Anaplasma* species in ruminants varies depending on geographic location, as well as tick habitat and animal care [48,49]. Moreover, different humidity levels that enable the proliferation of vectors and transmission of anaplasmosis can affect the prevalence rate [40].

From the results, it is clear that the age of the camels did not affect the prevalence of anaplasmosis and animals aged >2 to 5 years were at a higher risk than those aged >5 years. This finding is in contrast to those of Farooqi et al. [50], who reported that the age of the camels was a potential risk factor for the occurrence of anaplasmosis. In addition, Kocan et al. [51] observed that animals over the age of five are found to be less affected, which can be related to the fact that low-level infections over time lead to immunity against clinical anaplasmosis.

A further finding was that the sex of the animal was a significant variable for camel anaplasmosis, and females were more susceptible to infection than males. The findings contradict those of Azmat et al. [46], who found that male animals have higher infection rates than female animals. Our result is consistent with the findings of Maurizi et al. [52] and Belkahia et al. [53], who reported a higher prevalence rate in females in comparison with males. The differences between these findings can be explained since people in the area of the Javed study kept female camels for breeding purposes, so these animals performed few draft activities and were more exposed to tick infestation. Moreover, this is may be due to female immunosuppression, which can develop during pregnancy and lactation and has the potential to last two years [54].

Other factors that were found to be significant were the tick infestation of examined camels and whether acaricides were previously applied. Camels that were infested with ticks were at high risk of anaplasma infection compared with non-infested camels. Overall, these findings are in accordance with those reported by Atif [55], who reported that tick infestation made animals more susceptible to infection. We believe that Egyptian camels can be infested by a variety of ticks, particularly hard ticks, which are the main vector for *Anaplasma* spp. [56].

Furthermore, in line with the results of Azmat et al. [46], the current study found that good grooming practice significantly reduced the rate of *Anaplasma* spp. infection, because frequent grooming led to the early observation of ticks, which could be controlled immediately. Furthermore, grooming practises have a considerable impact on disease dynamics, which could be attributed to the fact that regular grooming practice allows for early detection of vectors and prompt control. In addition, the absence of grooming allowed the existence of hiding spots for vectors that are difficult to manage through farm manipulation. Similarly, emaciated camels were found to be more susceptible to infection, as was previously concluded by Azmat et al. [46]. This may be explained by the emaciation due to infestation with ticks and concurrent infection by other parasites or bacteria, which can increase the risk of infection [57].

The limitation of this study was the use of a commercial ELISA test for the detection antibodies against *A. marginale*, *A. centrale* and *A. ovis.* A specific test such as PCR is needed to determine the prevalent species among camels in Egypt. In addition, the present study is a cross-sectional study able to evaluate association only; therefore, longitudinal studies are required to prove causation.

## 5. Conclusions

It has been concluded that tick infestation, tick control status, grooming practice and body condition are strongly associated with *Anaplasma* spp. infection. In Egypt, the link between *Anaplasma* spp. infection and their arthropod vectors is mostly unknown, and more research is needed. However, further epidemiological and molecular studies are required to evaluate the situation of the disease across the country and to identify the genetic features of *Anaplasma* spp. in camels.

## Figures and Tables

**Table 1:** Risk factors associated with *Anaplasma* spp. infection in camels.

Factors	No of Examined Camels	No of Positive	%	95% CI	Statistic
Locality					
Qalyubia	115	23	20	13.7–28.2	χ^2^ = 2.956 *p* = 0.228
Kafr ElSheikh	100	13	13	7.7–20.9	
Red Sea	150	32	21.3	15.5–28.5	
Age					
≤2	43	8	18.6	9.7–32.6	χ^2^ = 0.744 *p* = 0.689
>2–5	210	42	20	15.2–25.9	
>5	112	18	16.1	10.4–23.9	
Sex					
Male	95	10	10.5	5.8–18.3	χ^2^ = 5.564 *p* = 0.018
Female	270	58	21.5	17–26.7	
Tick infestation					
Infested	115	38	33	25.1–42.1	χ^2^ = 23.009 *p* < 0.0001
Non-infested	250	30	12	8.5–16.6	
Application of acaricides					
Yes	140	15	10.7	6.6–16.9	χ^2^ = 9.388 *p* = 0.003
No	225	53	23.5	18.5–29.5	
Grooming Practice					
Applicable	180	20	11.1	7.3–16.5	χ^2^ = 13.245 *p* < 0.0001
Non-applicable	185	48	25.9	20.2–32.7	
Body condition					
Emaciated	120	51	42.5	34–51.4	χ^2^ = 67.194 *p* < 0.0001
Healthy	245	17	6.9	4.4–10.8	

The result is non-significant at *p* > 0.05. The result is significant at *p* < 0.05.

**Table 2:** Multivariable analysis of the potential risk variables for camel anaplasmosis.

Variable		B ^a^	SE ^b^	OR ^c^	95% CI ^d^	*p*-Value
Sex	Female	0.691	0.398	2.00	0.91–4.35	0.083
Tick infestation	Infested	0.116	0.371	1.12	0.54–2.32	0.755
Application of Acaricides	No	0.017	0.495	1.02	0.39–2.68	0.973
Grooming practice	non-applicable	0.260	0.457	1.30	0.53–3.18	0.570
Body condition	Emaciated	2.236	0.390	9.36	4.36–20.10	>0.0001

^a^ Logistic regression coefficient, ^b^ Standard error, ^c^ Odds ratio, ^d^ Confidence interval

## Data Availability

All data analyzed during this study are included in this published article.
